# Contemporary Chronic Limb-Threatening Ischemia Care in the United States—Part 1: A Path Toward Multispecialty Collaboration

**DOI:** 10.1016/j.jscai.2025.104013

**Published:** 2025-11-03

**Authors:** Eric A. Secemsky, Ehrin J. Armstrong, Venita Chandra, Raghu Kolluri, Parag J. Patel, Peter A. Schneider, Niten Singh

**Affiliations:** a Division of Cardiology, Beth Israel Deaconess Medical Center, Boston, Massachusetts; b Department of Interventional Cardiology, Advanced Heart and Vein Center, Denver, Colorado; c Division of Vascular Surgery, Stanford University Medical Center, Stanford, California; d Cardiovascular Service Line—OhioHealth Riverside Methodist Hospital, Columbus, Ohio; e Division of Interventional Radiology, Department of Radiology, Medical College of Wisconsin, Milwaukee, Wisconsin; f Division of Vascular and Endovascular Surgery, University of California, San Francisco, California; g Division of Vascular Surgery, Department of Surgery, Harborview Medical Center, Seattle, Washington

**Keywords:** chronic limb-threatening ischemia, cross-specialty, interdisciplinary, multispecialty, team-based care

## Abstract

Care for patients with chronic limb-threatening ischemia (CLTI) is complex, and it is most effective when conducted with collaboration across multiple specialties. A recent upward trend in major limb amputation among patients with CLTI warrants a renewed effort to optimize care for this multifaceted condition. The Vascular InterVentional Advances (VIVA) Foundation, a not-for-profit 501(c)(3) organization, convened a Vascular Leaders Forum in 2024 to initiate an open, multispecialty discussion about the state of CLTI care in the United States and current challenges around delivery and access to such care. The forum comprised representatives from vascular surgery, interventional cardiology, interventional radiology, vascular medicine, podiatry, regulators, medical device manufacturers, patient advocacy, and the CLTI and CLTI caregiver population. This article explores the central themes of challenges in CLTI care and ways in which collaboration across specialties and care settings could improve CLTI outcomes. In summary, it was recommended that integrated CLTI care teams extend beyond vascular surgery, interventional cardiology, and interventional radiology to include vascular medicine, podiatry, wound care, diabetology, and dietetics. Meeting the increasing demand for CLTI revascularization will require these teams to span tertiary care hospitals, community hospitals, outpatient revascularization clinics, and primary care settings.

## Introduction

The care of patients with chronic limb-threatening ischemia (CLTI) is multidimensional and is most effective when multiple specialties collaborate to optimize patient outcomes.^1^, ^2^, ^3^ Joint guidelines involving numerous specialty societies call for a combination of medical therapy, wound care, noninvasive and invasive vascular testing, and revascularization procedures to achieve limb salvage.^3^^,^^4^ As such, integrating care across medical specialties is recommended for successful treatment of individuals living with CLTI.

The Vascular InterVentional Advances (VIVA) Foundation convened a Vascular Leaders Forum on April 12, 2024, in Washington, DC, to initiate an open discussion exploring the state of CLTI care in the United States and current challenges around delivery and access to that care. The attendees comprised 52 US stakeholders, including representatives of vascular surgery (29%), interventional cardiology (26%), interventional radiology (24%), vascular medicine (7%), and podiatry (2%), as well as 2 US Food and Drug Administration representatives, 1 device manufacturer representative, 1 patient advocate, and 1 patient with CLTI and their caregiver. The practice settings were 70% academic tertiary care hospitals, 7% nonacademic community hospitals, 5% nonacademic tertiary care hospitals, and 5% office-based laboratories or ambulatory surgery centers. The list of attendees is available in Supplemental Table S1. This article summarizes the central themes of challenges in CLTI care and ways in which collaboration across specialties and care settings could improve CLTI outcomes.

## Challenges in contemporary CLTI care in the United States

Across the globe, the prevalence of peripheral artery disease (PAD) has been increasing continually over the past 3 decades.^5^, ^6^, ^7^ As the most severe form of PAD,^4^^,^^8^ CLTI is associated with significant mortality, limb loss, pain, health care costs ($10-$20 billion), and diminished quality of life.^4^^,^^8^, ^9^, ^10^, ^11^, ^12^ Approximately 25% to 30% of patients with CLTI undergo limb amputation within a year after diagnosis; CLTI's coexistence with diabetes makes it a global driver of amputation rates.^13^ After declining amputation rates in the United States from 2000-2010, a recent upward trend in major limb amputation among patients with CLTI is concerning.^14^^,^^15^ Despite the possibility for surgical or endovascular revascularization in most patients, amputation remains a first-line approach for many patients with CLTI who do not undergo an attempt at revascularization.^16^, ^17^, ^18^, ^19^, ^20^ A recent analysis of Medicare claims data from 2016-2019 found that a majority of patients with CLTI who underwent major lower limb amputation did not receive an angiogram or any attempt at revascularization before a nontraumatic CLTI-associated amputation.^21^

CLTI affects some of the most vulnerable members of our society. Racial and socioeconomic disparities in health care delivery influence clinical outcomes and are very much at play in the setting of CLTI. The burden of PAD disability and mortality has historically been, and remains to this day, elevated among African Americans; this includes lower rates of primary patency and limb salvage and higher rates of amputation compared with White patients.^22^, ^23^, ^24^, ^25^ Social determinants of health in PAD exist at both the individual and community levels, pointing to a complex interplay of factors that impacts disease severity, treatment, and outcomes.^26^^,^^27^ For example, data from the Vascular Quality Initiative shows that patients with PAD who live in neighborhoods with greater adversity and deprivation have more advanced disease at presentation as well as lower rates of revascularization.^27^

Disparities in access to CLTI care can also arise from financial barriers, lack of transportation, lack of patient awareness/knowledge of PAD, and variable intensity of vascular care across institutions.^17^ In the United States, limited access to CLTI care is reaching crisis levels, driven by an aging population, increasing PAD prevalence, and an insufficient supply of vascular specialists (due to retirement, too few trainees, and provider burnout).^28^ In the discipline of vascular surgery, 95% of vascular surgeons in the US practice in metropolitan areas, leaving relative vascular surgery deserts in both rural areas (1% of vascular surgeons) and strictly urban areas (4% of vascular surgeons).^28^

The financial strain on all health care settings, from hospitals to outpatient clinics, makes for a challenging CLTI reimbursement landscape, as Medicare reimbursement adjustments have not kept pace with inflation. In 2008, the Centers for Medicare and Medicaid Services (CMS) modified reimbursement rates to encourage performance of vascular procedures in outpatient settings in an effort to reduce costs and improve access.^29^ Rapid growth in the number of outpatient revascularization clinics followed.^30^ Come 2022, however, CMS had significantly reduced the Medicare physician fee schedule for interventionalists performing outpatient vascular interventional procedures and had lowered the valuation of devices, delivery systems, clinical labor, and supplies to the point where, today, reimbursement for revascularization is less than the procedure's direct costs.^31^ Outpatient revascularization offers patients greater access to care while providing physicians more autonomy with less bureaucracy. In the current reimbursement climate, however, this business model assumes pressure to perform more procedures as declining reimbursements impact financial viability.^32^, ^33^, ^34^

**Figure 1:**
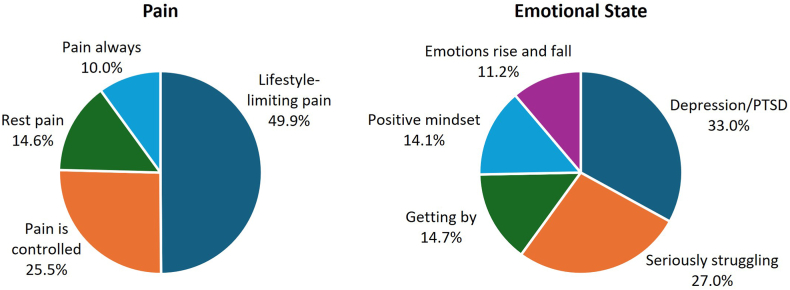
**Global Peripheral Artery Disease Association Facebook Poll (473 respondents).** PTSD, posttraumatic stress disorder.

Informal Facebook polls conducted by the Global PAD Association, although not based on science, provide a unique window into the challenges facing people living with CLTI. In these polls, patients report physical pain (often lifestyle-limiting) and emotional suffering as significant ramifications of their disease (Figure 1).^35^, ^36^, ^37^ They express wanting health care providers to acknowledge their pain and give them more therapeutic options that can ease their physical suffering and fortify their ability to walk. Patients also want their health care providers to explain the precise benefits of walking as a CLTI therapy rather than just telling them to “walk, walk, walk.” They also voice desire for options outside of amputation and assistance getting a second opinion, and many say that disparagement of other doctors (even if warranted) is unhelpful.^36^ In the Global PAD Association’s Leg Saver Hotline survey, most patients reported they were offered intervention for lifestyle-limiting claudication, rest pain, or ulcers. A significant number were offered first-line amputation and were told that amputation was inevitable.^38^ Many said they were first diagnosed with something other than PAD; some said they were not told they had PAD prior to amputation.^36^^,^^39^ Clearly, patients face challenges in getting an accurate diagnosis of PAD, understanding their disease, and being offered all treatment options before amputation; they also experience barriers to getting appointments, scheduling procedures, and obtaining insurance coverage.^35^, ^36^, ^37^^,^^39^ Additionally, patients say that conflicting opinions from different doctors are confusing and create a lack of trust. The fear endemic to distrust of health care professials and uncertainty about one's disease state contributes to mental health decline, including increased likelihood of anxiety and depression.^37^^,^^40^ From a medical perspective, a major factor influencing limb salvage is delay, whether due to fear or ignorance-driven patient delay in seeking care or lack of access to the health care system or a provider who understands the urgency of their condition.^35^^,^^37^^,^^38^

## Multispecialty collaboration in CLTI care

There is no one specialty that possesses the full skillset to provide optimal care for CLTI— no single speciality manages the gamut of surgical bypass, endovascular interventions, wound care, contemporary medical management, and glycemic control. Consequently, multispecialty collaboration to improve limb salvage involves a wide-ranging team that may include vascular surgeons, interventional radiologists, interventional cardiologists, vascular medicine specialists, cardiologists, podiatrists, wound care teams, diabetologists, and dietitians. In the absence of specialists fully dedicated to CLTI, team-based care is essential.

Multispecialty teams have the potential to improve the quality of care in today’s increasingly complex and fragmented health care delivery system.^41^ As has been previously alluded to, CLTI has many features that call for collaboration across medical specialties (Table 1).^42^ Multidiscipline, team-based care can facilitate more prompt diagnosis, smoother access to specialists, less variability in treatment practices, implementation of evidence-based treatment, discovery of novel solutions to complex issues, and a mechanism through which ongoing improvements can be made to the local CLTI care pathway. Team-based care models also help focus care planning and delivery on patients’ needs and can reinforce a shift away from fee-for-service payment to value-based payment that rewards improved safety, quality, and efficiency of health care delivery.

**Table 1:** Factors indicating a need for multispecialty care

Factors
Wide variations in treatment
Shared processes and protocols are needed
Lack of adequate evidence base to guide care
Complex/challenging/unusual cases
Careful coordination of care required
Patient ambivalence and poor health literacy
Poorly resourced patients and communities, in particular involving underrepresented minority patients

There are many examples of successful multispecialty team management for complex medical/surgical conditions that can serve as models for building a CLTI care team, such as heart valve teams, shock teams, stroke teams, pulmonary embolism response teams, and chronic thromboembolic pulmonary hypertension teams. A contemporary meta-analysis showed that in 31 of 33 analyzed studies, multispecialty team management of diabetic foot ulcers reduced major amputations for patients with diabetic foot ulcers.^43^ In the Netherlands, Brazil, Italy, Spain, United Kingdom, and Finland, major amputation rates have been shown to be lower in CLTI specialist centers that utilize team-based care, regardless of each country's brand of health care system.

Emerging evidence indicates that CLTI outcomes improve when multispecialty team care is implemented (Table 2) -- specifically, improvements in amputation-free survival^1^ and 1-year mortality with no increase in revascularization rate have been noted.^2^ A recent scoping review of efforts to address racial and ethnic disparities in PAD and/or CLTI care identified potential for improvement in this area through multidisciplinary care teams and algorithm-based treatment protocols that emphasize the importance of medication compliance, timely revascularization, and frequent follow-up (Table 2).^42^ One of the most notable efforts discussed in this review is the UCSF Fresno Comprehensive Heart and Multidisciplinary Limb Preservation Outreach Networks screening program, which aims to improve limb salvage rates in a rural region of California where access to vascular care is poor and Vascular Quality Initiative–trained providers are few. This program used desert mapping tools to target vascular testing to underserved areas and at-risk patients and characterize demographic characteristics and social factors impacting PAD care.^44^ Research has shown that primary care providers are unfamiliar with ankle-brachial index testing and the wound, Ischemia, and Foot Infection classification tool, and they will likely not be reimbursed for this point-of-care testing.^45^

**Table 2:** Examples of multispecialty team care and quality improvement initiatives in the PAD and CLTI settings

Focus	Model	Outcome
Initial and follow-up wound care from multidisciplinary team vs standard wound care in patients with CLTI^1^	Team of vascular, plastic, and podiatric surgeons jointly managed care, including weekly discussions, and directed other consults/services as deemed necessary	>2-fold improvement in amputation-free survival with multidisciplinary care team
Before and after launch of multidisciplinary limb salvage team for patients undergoing major lower extremity amputations^2^	Integrated limb salvage team consisting of vascular surgeons, podiatrists, plastic surgeons, advanced practice wound care providers, and internal medicine providers	Lower 1-y mortality and no increase in revascularization rate with multidisciplinary limb salvage team vs without
Scoping review of quality improvement efforts to address racial and ethnic disparities in PAD and/or CLTI^42^	Multidisciplinary team-based care, shared decision making, inpatient and outpatient care pathways, and revascularization goals	Comprehensive quality improvement protocol statement for PAD
	QI program with 4 process measure targets: hospital admission ≤2 d of referral, imaging ≤12 h of admission, examination by vascular specialist ≤14 h of admission, and limb revascularization ≤5 d of admission	Increase in meeting all stated targets
	Quality of care score that assessed compliance with 4 medical management components: antiplatelet therapy, dyslipidemia management, hypertension control, and glucose control in diabetes	Non-Hispanic White and Black patients more likely to have higher quality of care scores than Hispanic patients
	Compliance with annual lipid and HbA1c testing among diabetic patients with CLTI undergoing lower extremity intervention	Higher annual testing compliance associated with lower MALE rates and better amputation-free survival, although Black patients fared worse on both outcomes
	Quality improvement program to identify undiagnosed PAD among Black men: outreach, awareness, screening, and education	Improved awareness of PAD and PAD-specific care process, identified undiagnosed PAD in one-fifth of participants
	Impact of statewide expanded insurance coverage on disparities in access to care between White and non-White patients with PAD	Reduced gap between White and non-White patients presenting with severe disease and significantly increased their chances of undergoing revascularization surgery

CLTI, chronic limb-threatening ischemia; MALE, major adverse limb event; PAD, peripheral artery disease.

## Multispecialty, team-based CLTI care

**Figure 2:**
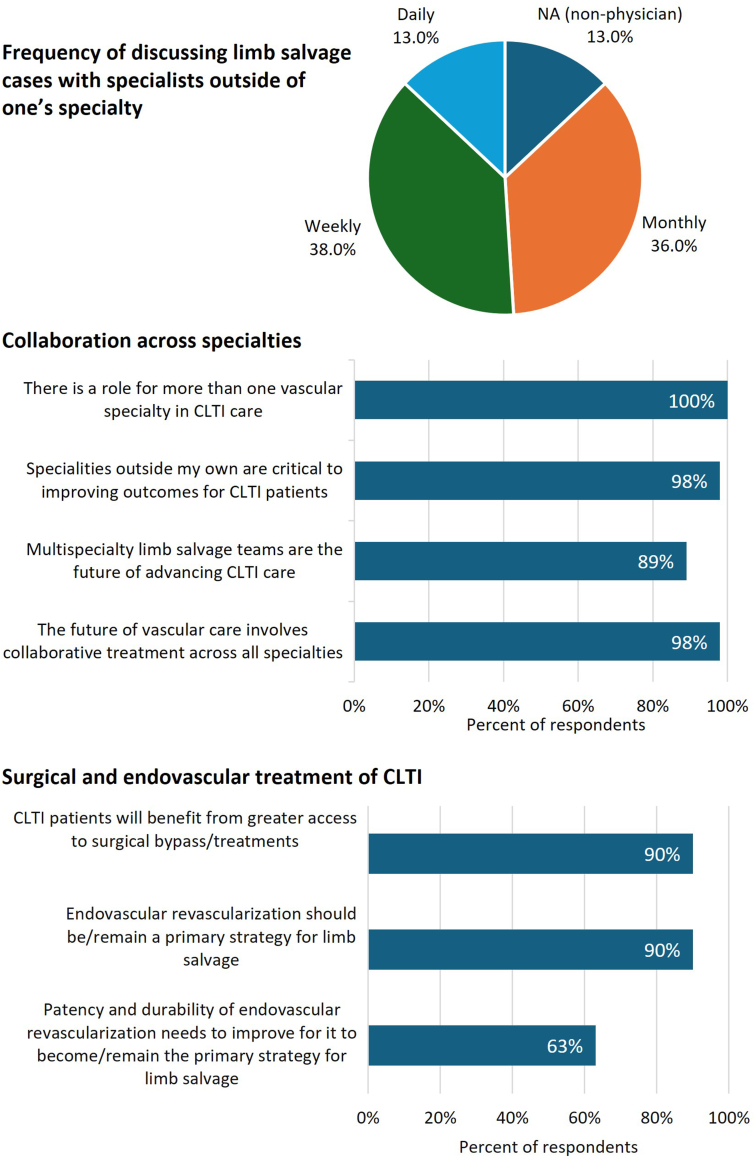
**Perspectives on cross-specialty collaboration among VIVA Vascular Leader Forum attendees.** CLTI, chronic limb-threatening ischemia.

Encouragingly, there are signs that seeds of collaboration among CLTI providers have been sown and are beginning to sprout. An ad hoc poll of VIVA Vascular Leaders Forum attendees showed that nearly all respondents believe collaboration across multiple specialties is needed to provide optimal CLTI care; most indicated that they discuss limb salvage cases with a colleague from another specialty monthly or weekly, some even daily (Figure 2). Nearly all voiced that both surgical and endovascular treatments are essential and that limb salvage specialty care teams are important to the future of CLTI care (Figure 2). However, some philosophic differences were noted among respondents. For instance, 63% expressed belief that patency and durability of endovascular intervention needs to improve for the revascularization modality to be considered a primary strategy for limb salvage (Figure 2).

It was recommended that integrated CLTI care teams extend beyond vascular surgery, interventional cardiology, and interventional radiology to include vascular medicine, podiatry, wound care, diabetology, and dietetics (Central Illustration). Meeting the increasing demand for CLTI revascularization will require that these multidisciplinary teams span tertiary care hospitals, community hospitals, outpatient revascularization clinics, and primary care settings.

**Central Illustration:**
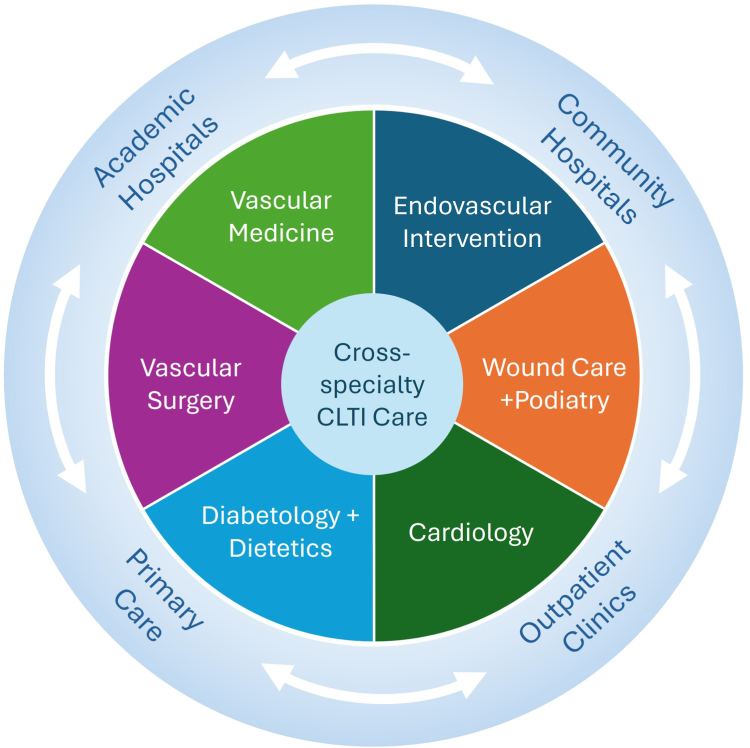
**Integrated CLTI care across specialties and care settings.** CLTI, chronic limb-threatening ischemia.

Building teams of health care providers to deliver integrated CLTI care across diverse care settings and institutions will not be simple or rapid. It will require institutional and regulatory changes, including changes in reimbursement structure, and culture shifts. Data collection will be needed to understand how different care settings approach CLTI care. Innovative “patient care pathway” models must be created and tested to accommodate diverse skill setsand organizational structures at different institutions in varied care settings. Ultimately, multispecialty, team-based CLTI care will require a set of mutually agreed-upon principles andprotocols akin to the Center of Excellence model at academic tertiary care centers. These may include a concrete definition of rest pain, indications for intervention in mild ischemia (eg, toe pressure of >80 mm Hg), optimal timing for intervention, and agreed-upon anatomical criteria to consider surgical revascularization as the firstline approach.

Fostering collaboration among specialists in CLTI care will need to be multifactorial. Health care providers may begin cultivating a norm of collaboration by increasing the frequency and quality of cross-specialty case discussions and by participating in quality metrics registries. Principal investigators can incorporate multispecialty CLTI care into the design of clinical trials of CLTI interventions. On an institutional level, it will be necessary to evaluate and harmonize the strengths and skills of each specialty, organization, and care setting.

In this article, we take a moment to envision a future where multispecialty team-based CLTI care is standard. Ideally, widespread PAD screening would become a reality through garnering support from the United States Preventive Services Task Force, training primary practitioners in ankle-brachial index measurement, and convincing health insurers to cover these assessments in the primary care setting. Surgeons, interventionalists, vascular medicine physicians, podiatrists, and wound care specialists would consult regularly on CLTI patients, particularly more complex cases. This team, along with diabetologists and dietitians, would holistically review these patients and aim to address all clinical factors contributing to PAD. The core CLTI care team might share the same physical space to enhance the sharing of ideas and hold regular virtual team meetings with members at other locations. A team-based environment of this kind promises to be rich in novel ideas for improving the lives of individuals with PAD. Implementation of this vision will require actionable, evidence-based guidelines for CLTI care team formation; consensus on strategies for early PAD detection and prevention; and evaluation of long-term outcomes of multispecialty team CLTI care and cost-effectiveness of integrated care models.

## References

[1] Chung J., Modrall J.G., Ahn C., Lavery L.A., Valentine R.J. (2015). Multidisciplinary care improves amputation-free survival in patients with chronic critical limb ischemia. J Vasc Surg.

[2] Kawaji Q., Martinson J., Husain S. (2025). Multidisciplinary limb salvage care is associated with decreased mortality without increasing revascularization in major amputations. J Vasc Surg.

[3] Gornik H.L., Aronow H.D., Goodney P.P. (2024). 2024 ACC/AHA/AACVPR/APMA/ABC/SCAI/SVM/SVN/SVS/SIR/VESS guideline for the management of lower extremity peripheral artery disease: a report of the American College of Cardiology/American Heart Association Joint Committee on Clinical Practice Guidelines. Circulation.

[4] Conte M.S., Bradbury A.W., Kolh P. (2019). Global vascular guidelines on the management of chronic limb-threatening ischemia. J Vasc Surg.

[5] GBD 2019 Peripheral Artery Disease Collaborators (2023). Global burden of peripheral artery disease and its risk factors, 1990-2019: a systematic analysis for the Global Burden of Disease Study 2019. Lancet Glob Health.

[6] Eid M.A., Mehta K., Barnes J.A. (2023). The global burden of peripheral artery disease. J Vasc Surg.

[7] Song P., Rudan D., Zhu Y. (2019). Global, regional, and national prevalence and risk factors for peripheral artery disease in 2015: an updated systematic review and analysis. Lancet Glob Health.

[8] Kwong M., Rajasekar G., Utter G.H., Nuno M., Mell M.W. (2023). Updated estimates for the burden of chronic limb-threatening ischemia in the Medicare population. J Vasc Surg.

[9] Duff S., Mafilios M.S., Bhounsule P., Hasegawa J.T. (2019). The burden of critical limb ischemia: a review of recent literature. Vasc Health Risk Manag.

[10] Eid M.A., Mehta K.S., Goodney P.P. (Mar 2021). Epidemiology of peripheral artery disease. Semin Vasc Surg.

[11] Nehler M.R., Duval S., Diao L. (2014). Epidemiology of peripheral arterial disease and critical limb ischemia in an insured national population. J Vasc Surg.

[12] Popplewell M.A., Andronis L., Davies H.O.B. (2022). Procedural and 12-month in-hospital costs of primary infra-popliteal bypass surgery, infrapopliteal best endovascular treatment, and major lower limb amputation for chronic limb threatening ischemia. J Vasc Surg.

[13] Norgren L., Hiatt W.R., Dormandy J.A. (2007). Inter-Society Consensus for the Management of Peripheral Arterial Disease (TASC II). J Vasc Surg.

[14] Creager M.A., Matsushita K., Arya S. (2021). Reducing nontraumatic lower-extremity amputations by 20% by 2030: time to get to our feet: a policy statement from the American Heart Association. Circulation.

[15] Geiss L.S., Li Y., Hora I., Albright A., Rolka D., Gregg E.W. (2019). Resurgence of diabetes-related nontraumatic lower-extremity amputation in the young and middle-aged adult U.S. population. Diabetes Care.

[16] Goodney P.P., Travis L.L., Nallamothu B.K. (2012). Variation in the use of lower extremity vascular procedures for critical limb ischemia. Circ Cardiovasc Qual Outcomes.

[17] Henry A.J., Hevelone N.D., Belkin M., Nguyen L.L. (2011). Socioeconomic and hospital-related predictors of amputation for critical limb ischemia. J Vasc Surg.

[18] Mustapha J.A., Katzen B.T., Neville R.F. (2018). Determinants of long-term outcomes and costs in the management of critical limb ischemia: a population-based cohort study. J Am Heart Assoc.

[19] Mustapha J.A., Katzen B.T., Neville R.F. (2018). Disease burden and clinical outcomes following initial diagnosis of critical limb ischemia in the medicare population. JACC Cardiovasc Interv.

[20] Valle J.A., Waldo S.W. (2018). Worth an arm and a leg: the critical importance of limb ischemia. J Am Heart Assoc.

[21] Secemsky E.A., Kirksey L., Quiroga E. (2024). Impact of intensity of vascular care preceding major amputation among patients with chronic limb-threatening ischemia. Circ Cardiovasc Interv.

[22] Rowe V.L., Weaver F.A., Lane J.S., Etzioni D.A. (2010). Racial and ethnic differences in patterns of treatment for acute peripheral arterial disease in the United States, 1998-2006. J Vasc Surg.

[23] Wahood W., Duval S., Takahashi E.A., Secemsky E.A., Misra S. (2023). Racial and ethnic disparities in treatment of critical limb ischemia: a national perspective. J Am Heart Assoc.

[24] Arya S., Binney Z., Khakharia A. (2018). Race and socioeconomic status independently affect risk of major amputation in peripheral artery disease. J Am Heart Assoc.

[25] Krawisz A.K., Natesan S., Wadhera R.K. (2022). Differences in comorbidities explain Black-White disparities in outcomes after femoropopliteal endovascular intervention. Circulation.

[26] Kassavin D., Mota L., Ostertag-Hill C.A. (2024). Amputation rates and associated social determinants of health in the most populous US counties. JAMA Surg.

[27] Mota L., Marcaccio C.L., Zhu M. (2023). Impact of neighborhood social disadvantage on the presentation and management of peripheral artery disease. J Vasc Surg.

[28] Potluri V.K., Bilello J.L., Patel S.G., Yarra S., Sykes M.T., Silva M.B. (2023). Characterizing the geographic distribution of vascular surgeons in the United States. J Vasc Surg.

[29] Centers for Medicare & Medicaid Services (CMS), HHS (2007). Medicare program; prospective payment system for long-term care hospitals RY 2008: annual payment rate updates, and policy changes; and hospital direct and indirect graduate medical education policy changes. Final rule. Fed Regist.

[30] U.S. Office-Based Labs (OBLs) Market Analysis 2018-2023 Grand View Research, Inc. https://www.grandviewresearch.com/industry-analysis/office-based-labs-obl-market/request/rs1.

[31] Blebea J., Jain K., Cheng C.I., Pittman C., Daugherty S. (2023). Expected changes in physician outpatient interventional practices as a result of coronavirus disease 2019 and recent changes in Medicare physician fee schedule. J Vasc Surg Venous Lymphat Disord.

[32] Itoga N.K., Baker L.C., Mell M.W. (2019). Impact of office-based laboratories on physician practice patterns and outcomes after percutaneous vascular interventions for peripheral artery disease. J Vasc Surg.

[33] Brown C.S., Smith M.E., Kim G.Y. (2021). Exploring the rapid expansion of office-based laboratories and peripheral vascular interventions across the United States. J Vasc Surg.

[34] Hicks C.W. (2022). Atherectomy overuse is a real problem. J Vasc Surg.

[35] Global PAD Association (2024). PAD Care in 2023-2024. Have you noticed a difference in care since the article came out last summer? Facebook poll. Posted March.

[36] McNicholas K. (April 2021). https://www.facebook.com/groups/peripheralarterialdisease/permalink/4542245469123789.

[37] McNicholas K.Y. (April 2021). https://www.facebook.com/groups/peripheralarterialdisease/permalink/4542245469123789.

[38] Global PAD Association (Posted March 13, 2023). PAD Treatment for Intermittent Claudicants vs. CLI. There is a big debate over whether physicians are doing surgery too soon, too late or not at all. Please weigh-in and also share your story in comments to support your vote. Facebook poll.

[39] Global PAD Association (Posted April 19, 2021). PAD Diagnosis. Before PAD was officially diagnosed, my doctor brushed off my symptoms of leg pain, cramping, and trouble walking as: Facebook poll. https://www.facebook.com/groups/peripheralarterialdisease/permalink/4507709705910699/.

[40] Global PAD Association (Posted November 23, 2023). I have to do a presentation for doctors on why people with PAD fall through the cracks. Can you help with lending your voice? Facebook poll. https://www.facebook.com/groups/peripheralarterialdisease/permalink/7581125865235719/.

[41] Schmutz J.B., Meier L.L., Manser T. (2019). How effective is teamwork really? The relationship between teamwork and performance in healthcare teams: a systematic review and meta-analysis. BMJ Open.

[42] Musuuza J., Sutherland B.L., Kurter S., Balasubramanian P., Bartels C.M., Brennan M.B. (2020). A systematic review of multidisciplinary teams to reduce major amputations for patients with diabetic foot ulcers. J Vasc Surg.

[43] Howell C., Brooke B.S. (2024). Quality improvement efforts to address racial and ethnic disparities in patients with peripheral vascular disease and chronic limb-threatening ischemia. JVS Vasc Insights.

[44] DiLosa K.L., Nguyen R.K., Brown C., Waugh A., Humphries M.D. (2023). Defining vascular deserts to describe access to care and identify sites for targeted limb preservation outreach. Ann Vasc Surg.

[45] DiLosa K., Brown C., Rajasekar G., Nuno M., Humphries M.D. (2023). Provider ankle brachial index and wound classification teaching as part of a comprehensive limb preservation outreach program. J Vasc Surg.

